# Sorption of Heavy Metal Cations on Mesoporous ZSM-5 and Mordenite Zeolites

**DOI:** 10.3390/ma12193271

**Published:** 2019-10-08

**Authors:** Kamila M. Wojciechowska, Magdalena Król, Tomasz Bajda, Włodzimierz Mozgawa

**Affiliations:** 1 Faculty of Materials Science and Ceramics, Department of Silicate Chemistry and Macromolecular Compounds, AGH University of Science and Technology, Al. Mickiewicza 30, 30-059 Krakow, Poland; mkrol@agh.edu.pl (M.K.); mozgawa@agh.edu.pl (W.M.); 2 Faculty of Chemistry, Jagiellonian University in Kraków, Gronostajowa 2, 30-387 Krakow, Poland; 3 Faculty of Geology, Geophysics and Environmental Protection, Department of Mineralogy, Petrography and Geochemistry, AGH University of Science and Technology, Al. Mickiewicza 30, 30-059 Krakow, Poland; bajda@agh.edu.pl

**Keywords:** mordenite, ZSM-5, mesoporosity, sorption properties, heavy metal cations, IR spectroscopy

## Abstract

Desilication and dealumination techniques were used to obtain mesoporous ZSM-5 and mordenite zeolites. The study provides insight into specific structural, textural, and sorption properties of obtained materials with different Si/Al ratios. Subsequent dealumination and desilication procedures were found to be efficient methods of generating a secondary system of mesopores in mordenite and ZSM-5 crystals while preserving their microporous character. The investigated materials were evaluated in terms of their sorption properties of selected heavy metal cations (Cd^2+^, Cr^3+^, and Pb^2+^). Particular emphasis was placed on the structural examination of the materials via infrared spectroscopy (FT-IR). Other research methods included X-ray diffraction (XRD), X-ray fluorescence (XRF), nuclear magnetic resonance (NMR) and atomic absorption spectrometry (AAS).

## 1. Introduction

Zeolites that belong to tecto-aluminosilicates are crystalline, nanoporous materials built of a three-dimensional framework of superimposed aluminate [AlO_4_] and silicate [SiO_4_] tetrahedra linked by shared oxygen atoms. Owing to their good sorption and ion-exchange properties as well as thermal and chemical stability, zeolites are successfully used in environmental protection and in many industries [1,2]. The structure of zeolite materials provides them with a high cation-exchange capacity and the ability to sorb harmful molecules or ions, e.g., Ag^+^, Cu^2+^, Ni^2+^, Zn^2+^, Cd^2+^, Pb^2+^, or Cr^3+^ [3].

Mordenite (MOR) is a rarely encountered natural mineral with a chemical composition expressed by the formula Na_8_[Al_8_Si_40_O_96_]∙24H_2_O [4]. The Si/Al modulus of mordenite equals approximately 5, but depending on its origin and the conditions of its creation it differs within a narrow range, whereas the exchangeable cations are mostly sodium, rarely potassium. Mordenite is part of the *Cmcm* space group, crystallizes in an orthorhombic system, and has lattice parameters equal to: *a* = 18.256 Å, *b* = 20.534 Å, and *c* = 7.542 Å [5]. Two types of perpendicular channels may be found in the space lattice of mordenite: Elliptical channels (12-member rings) parallel to the [001] direction with the size of 7.0 Å × 6.7 Å as well as parallel to the [010] direction with the size of 5.7 Å × 2.6 Å (eight-member rings) [6]. Additionally, the framework includes other channels with an eight-member ring cross-section, connected to big channels, parallel to the [010] direction, with sizes of 4.8 Å × 3.4 Å [7]. Both types of channels are running independently and do not cross; thus, mordenite is considered a zeolite with a one-dimensional system of channels. Due to the particular system of pores and lack of a direct connection between neighboring 12-member channels, there is a possibility of diffusion of large cations or molecules in only one direction. It is worth mentioning that the structure of zeolite consists of four- and five-member rings as well, which are interconnected by a common oxygen atom. Basing on the 5-1 secondary building units, this zeolite is part of the pentasil group. Mordenite finds a great range of applications as a catalyzer for many significant catalytic reactions, e.g., hydrocracking or hydroisomerization.

The ZSM-5 zeolite, due to its high specific surface area, great shape selectiveness, acidity, and high hydrothermal stability, is one of the most important heterogeneous catalyzers, highly used in catalytic reactions, e.g., cracking, isomerization, alkylation [8]. This material is part of the 5-1 pentasil group and is characterized by a silicon modulus of above 10. The ZSM-5 zeolite belongs to the MFI (framework type MFI from ZSM-5 (five)) group, with a general composition of M_xn_[(AlO_2_)_x_(SiO_2_)_96−x_]∙16H_2_O, *Pnma* space group and lattice parameters of: *a* = 20.090 Å, *b* = 19.738 Å, and *c* = 13.142 Å [5]. The structure of the zeolite consists of TO_4_ (T = Si or Al) tetrahedrons, connected into five-member rings, which create a three-dimensional system of pores. Among them we may distinguish straight elliptical channels, running along the [100] direction, with sizes of 5.1 Å × 5.6 Å and sinusoidal, running along the [010] direction, with the size of 5.3 Å × 5.6 Å. The result of their intersecting are chambers, 5.4 Å in size. The zeolite crystallizes in an orthorhombic system.

The immobilization of heavy metal cations involves their total deactivation and binding in the zeolite structure. Many research groups intensively studied the issue of sorption of heavy metals on zeolites [9,10,11]. The cation-exchange capacity of aluminosilicates is one of their most useful properties, allowing them to be used for heavy metal removal. Although this process involves adsorption, chemisorption (understood as the chemical phenomena, such as surface precipitation, etc.), and ion exchange, it is predominantly the last of these phenomena that is responsible for the high sorption capacity of these materials [12]. The sorption capacity of zeolites has a tendency to decrease with increasing concentration of heavy metals in water, and their selectivity is strongly affected by pH and the type of cation [9]. Literature data [3] indicate that in the case of the sorption of lead(II) and chromium(III) by clinoptilolite, chemisorption is the dominant sorption mechanism, while in the case of nickel(II) and cadmium(II), ion-exchange and chemisorption both contribute to the same degree. On the other hand [11], investigations of the sorption of Pb^2+^, Ni^2+^, Zn^2+^, and Cd^2+^ cations by means of clinoptilolite and erionite have proven the higher sorption efficiency of erionite, which exhibited the following selectivity: Pb^2+^ > Ni^2+^ > Zn^2+^ > Cd^2+^ [13].

Heavy metals pose a serious threat to the natural environment, and thus to human health [14,15,16]. Heavy metals get into the human body from soil, air, and water. Lead naturally occurs in water in a small amount—in the natural environment it is mainly present in the form of sparingly soluble salts. Increased lead content in plants contributes to the disturbance of photosynthesis [17]. Lead toxicity to the human body is manifested in damage to the nervous system. Predominantly deposited in the bones, it has a negative effect on the circulatory, nervous, respiratory, kidney, and liver functions [15,18]. Chromium in the natural environment most often occurs in the form of the Cr^3+^ cation, which is characterized by easy bioaccumulation. Chromium on the VI oxidation state is a carcinogen; however, anions CrO_4_^2−^ and Cr_2_O_7_^2−^ more easily penetrate the human body than Cr^3+^ cations [15]. These salts easily enter the bloodstream. Chromium has a negative effect on the digestive system, and its presence in the body can cause changes on the skin. Excessive cadmium accumulation in plants negatively affects the photosynthesis process and may even lead to changes in DNA structures [17]. It has a negative effect on the excretory, respiratory, and circulatory systems [17].

The main focus of the presented work was the modification of the structures of mordenite and ZSM-5 zeolites, performed in order to improve sorption properties. To improve these properties, a number of modifications to their structure have been used, based on the generation of a secondary mesopores system while maintaining the microporous nature of the materials. The proposed modification was dictated by the fact that in the case of nanoporous materials, access to active sites and ion transport is severely difficult. The actual sorption capacity of zeolite may be lower than the theoretical value. The attempt to obtain effective systems that allow free diffusion was carried out. The concept of hierarchical zeolites proved to be an effective method of improving the sorption properties of zeolites. Sorption capacity increased. Modification involved both desilication and dealumination. The effectiveness of the method was evaluated by sorbing selected heavy metal cations: Cd^2+^, Cr^3+^, and Pb^2+^. IR spectroscopy was used in order to determine the sorption properties of the materials before and after modification. Based on the obtained spectra, the zeolite structure was described in detail taking into account the manner and degree of modification as well as their sorption properties.

## 2. Materials and Methods

### 2.1. Material Preparation

Parent zeolite materials (Na-mordenite: Si/Al = 8, Zeolyst: CBV 10A and NH_4_-ZSM-5: Si/Al = 25, Zeolyst) were modified in order to obtain mesoporous materials. According to the producer website Zeolyst, the particle size of ZSM-5 zeolite and mordenite is: 0.5–2 μm. Three techniques were applied for this purpose: (i) modification using a NaOH hydroxide solution, (ii) modification using an HNO_3_ acid solution, and (iii) a combination of methods (i) and (ii). The obtained samples received the designation MOR/ZSM(MA_C_MA__T_t), where C_MA_ was the concentration of modification agent, MA was the modification agent (NaOH or HNO_3_), T and t represented the temperature and time of modification process, respectively. For example, a mordenite sample treated with a 2 M HNO_3_ solution at 85 °C for 1 h and then with a 0.2 M NaOH solution at 65 °C for 0.5 h is referred to as MOR(HNO_3__2_85_1/NaOH_0.2_65_0.5). In the case of ZSM-5 zeolite, one method of modification was used because this method turned out to be the most effective [19].

Each modification started with the introduction of a zeolite sample and acid or basic solution into a round-bottom flask (100 mL of solution per 3 g of zeolite), followed by heating using an oil bath. After the appropriate amount of time had elapsed, samples were cooled in an ice bath, filtered, and rinsed three times with distilled water. In the next step, aimed to obtain H^+^-forms, modified samples and parent Na-mordenite sample underwent a threefold ion exchange with 0.5 M of an NH_4_NO_3_ solution (T = 60 °C, t = 1 h)—sample noted as MOR_P. The ZSM-5 zeolite, which was in the NH_4_^+^-form, was omitted from this part of the procedure (ZSM_P). Finally, all samples were filtered and rinsed with distilled water and dried at room temperature. The samples prepared in this way were subsequently calcined at 450 °C for 3 h (mordenite) and at 500 °C for 2 h (ZSM-5), yielding the H^+^-forms of the investigated zeolites. Each sample were transformed into H^+^-form during the calcination process.

All prepared samples are listed in Table 1.

For sorption studies, parent ZSM_P and MOR_P, as well as the modified materials designated as MOR (HNO_3__2_85_1/NaOH_0.2_65_0.5) and ZSM (NaOH_0.2_65_0.5), were selected. The selected samples were used for the sorption of heavy metal cations (Pb^2+^, Cd^2+^, and Cr^3+^) with the use of aqueous solutions of the proper nitrates(V) salts. For each cation, solutions containing initial concentrations of 1, 2, 5, 15, and 20 mmol/dm^3^ were prepared. Heavy metal solutions were prepared in triple distilled water. For each of these concentrations, sorption was carried out in parallel on four samples of each of the studied zeolites. During the sorption process, the suspension of a given zeolite with a solution of the suitable salt (at a solid-to-liquid phase weight ratio of 1:50) was shaken for 24 h. The sorption experiments were performed at a speed of 1000 rpm. Sorption processes were carried out at room temperature. The samples were subsequently centrifuged, and the solution was collected, leaving the precipitate. In order to determine the contribution of particular mechanisms involved in the sorption of heavy metal cations, i.e., ion exchange and chemisorption, their desorption was carried out. The samples were treated with a 2 M NaCl solution and then shaken and centrifuged in the same manner as in the sorption process. The concentrations of metal cations in solutions before and after sorption and desorption were determined via atomic absorption spectroscopy (AAS-SavantAA GBC Scientific Equipment, Braeside, Australia).

### 2.2. Measurement Methodology

A fluorescence spectrometer (WDXRF, Axios Max 4 kW, PANalytical, Almelo, The Netherlands) was used to analyze the chemical composition of parent zeolite materials and their modified counterparts. The obtained results were presented on a percentage scale (normalized to 100%). In addition, the Si/Al ratios of the analyzed materials were determined. The chemical compositions of all formulations are shown in Table 2.

The concentrations of heavy metal cations in solutions after sorption and desorption were determined using atomic absorption spectroscopy (AAS-SavantAA GBC Scientific Equipment, Braeside, Australia), based on the calibration curve. The measurement of ion concentration in the solution after the desorption process allowed us to determine the number of ions that underwent ion exchange, while the percentage of chemisorptions has been determined using the difference between the number of cations after full sorption and the concentration after desorption. The percentage of ion exchange is understood as the quotient of the desorption value (mmol/kg) and the total sorption value (mmol/kg). The result was normalized to 100%. In turn, the percentage of chemisorptions is the difference between the total sorption (100%) and the percentage of ion exchange (Equation (1)).

% chemisorption = 100% − % ion-exchange(1)

Powder X-ray diffraction (XRD) measurements were carried out using an X’Pert diffractometer (PANalytical, Almelo, The Netherlands), with the use of CuKα radiation, a wavelength of 1.5406 Å, an intensity of 30 mA, and a graphite monochromator. Measurements were carried out in the 2θ angle range of 10–60° for 4 h, with a step of 0.008. The aim of this procedure was to determine the degree of crystallinity (understood as degree of structural order in a solid) of the samples, based on the intensity of the characteristic peaks [20] for mordenite and [21,22] for the ZSM-5 zeolite) in the 2θ range of 20–24°. The obtained results have been shown on a percentage scale.

The standard procedure and the BET (Brunauer, Emmett and Teller isotherm) isotherm equation in the adsorption of nitrogen vapors have been used. Low-temperature adsorption and desorption of N_2_ experiments were conducted at −196 °C using the ASAP 2000 (Micromeritics Instrument Corporation, Norcross, USA). Measurements were preceded by 12 h of activation under vacuum at 370 °C. The surface area (S_BET_) and micropore volume (V_micro_) were determined using BET and *t*-plot. BJH adsorption isotherms were used to establish the volume of mesopores (V_meso_).

The infrared spectra of parent and modified materials were measured with a Bruker VERTEX 70v vacuum spectrometer (Bruker Optics Inc., Billerica, MA USA). For this purpose, KBr (Merck) pellets were prepared and mid-infrared (MIR) spectra were measured. The spectra were recorded by performing 128 scans at a resolution of 4 cm^−1^.

NMR spectra were obtained using APOLLO (Tecmag, Houston, Texas) in a 7.05 T magnetic field (Magnex). For ^29^Si MAS NMR spectra, a pulse of 3 μsrf (angle of π/2), a centrifugation speed of 4 kHz, and 256 scans with 40 s of delay were applied. Before recording ^27^Al MAS NMR spectra, the samples were kept at a relative humidity of 75% for 48 h. The spectra were recorded using a pulse of 2 μsrf (angle of π/6), spin speed 8 kHz and 1000 scans with a delay of 1 s. The chemical shift was reported in ppm, with respect to TMS and a 1 M Al(NO_3_)_3_ solution for ^29^Si and ^27^Al MAS NMR spectra, respectively. NMR spectra were normalized to the same sample mass.

## 3. Results and Discussion

### 3.1. Chemical and Phase Composition of the Studied Samples

The obtained results, including Si/Al ratio, degree of crystallinity, and textural parameters, are presented in Table 3.

The aluminum content in the zeolite structure had a significant influence on the silicon extraction process. This is particularly evident in the case of mordenite. The direct desilication of parent material with sodium hydroxide did not significantly affect the Si/Al ratio, which indicates that the amount of removed silicon was small and the efficiency of this process was low. This is related to the high aluminum content in the mordenite structure because the presence of [AlO_4_] tetrahedra, negatively charged due to electrostatically repelling OH^−^ ions, stabilizes silicon atoms in the immediate vicinity, which effectively prevents desilication. Similar observations were noted for the ZSM-5 zeolite treated with the sodium hydroxide solution. The performed modification led to a decrease in the Si/Al ratio—down to 24.

The treatment of mordenite with the 2 M HNO_3_ solution doubled its Si/Al value. Similarly, in the case of mordenite dealuminated with 4 M HNO_3_ solution, the silicon to alumina ratio increased almost threefold. The obtained values of Si/Al indicate excellent efficiency of extraction of aluminum from the structure of tested materials. In addition, it is worth noting that the desilication of thus modified materials resulted in a decrease in Si/Al ratios compared to previously dealuminated materials. In these cases, an increased amount of removed silicon correlated with a significantly lower content of aluminate tetrahedra, which stabilize the zeolite structure.

**Figure 1:**
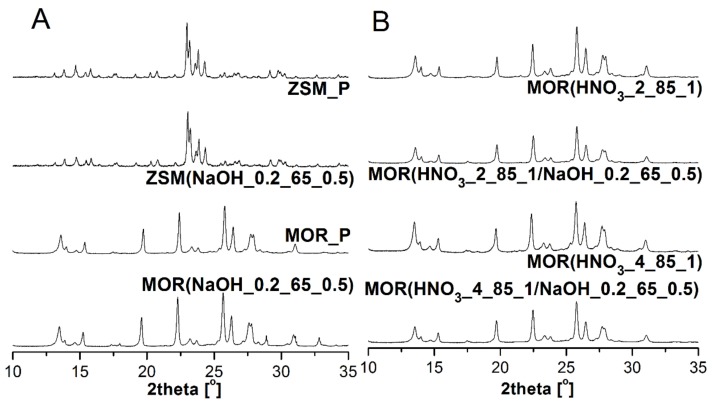
XRD patterns of parent and modified zeolite materials: A: ZSM-5 and B: mordenite.

The applied type of modifications is occasionally associated with a decrease in degree of crystallinity and even the amorphization of materials [23,24]. Figure 1 shows the X-ray diffraction patterns of parent and modified materials.

In the case of ZSM(NaOH_0.2_65_0.5), no significant changes in diffraction patterns were observed. The modified material retained its crystalline character, as evidenced by the determined degree of crystallinity (94%). In the case of mordenite and the direct treatment of parent material with the sodium hydroxide solution, crystallinity dropped slightly, even though the amount of removed silicon was minimal. It is worth noting that dealumination performed in two different ways and the subsequent desilication caused only a slight decrease in a degree of crystallinity. This is particularly evident in the case of MOR treated with a 2 M HNO_3_ solution and then with 0.2 M NaOH.

### 3.2. Textural Studies

Table 3 presents the results of textural studies, including the specific surface area (S_BET_), external surface area (S_EXT_) as well as micro- (V_micro_) and mesopore (V_meso_) volumes. Parent materials (ZSM_P and MOR_P) are characterized by micropore volumes that are typical of their structures, namely 0.18 and 0.17 cm^3^/g, respectively. The effectiveness of the demetalation processes was determined based on the changes in the textural properties. The treatment of MOR_P with sodium hydroxide solution did not affect either the specific surface area or the micropore volume. Likewise, mesopore volume did not significantly increase with respect to parent material (Table 3). It can therefore be concluded that desilication was ineffective. On the other hand, the analysis of the results obtained for MOR(HNO_3__4_85_1) revealed that the volume of mesopores (V_meso_ = 0.166 cm^3^/g) hardly changed compared to that of MOR_P. The modification of the dealuminated zeolite with the NaOH solution resulted in a significant increase in mesopore volume, and a decrease in BET surface area and micropore volume. The generation of a secondary mesopore system (the simultaneous presence of micro- and mesopores) in the case of MOR(HNO_3__2_85_1/NaOH_0.2_65_0.5) is confirmed by the increased specific surface area and mesopore volume (V_meso_ = 0.292 cm^3^/g). This is consistent with XRD results. In the case of mordenite, it is known that there is a larger share of aluminum than in high-silica zeolites, hence initial dealumination proved to be an indispensable stage of modification.

The desilication of parent ZSM_P material with sodium hydroxide resulted in an increase in S_BET_ and V_meso_, while the micropore volume remained virtually unchanged (V_micro_ = 0.17 cm^3^/g).

**Figure 2:**
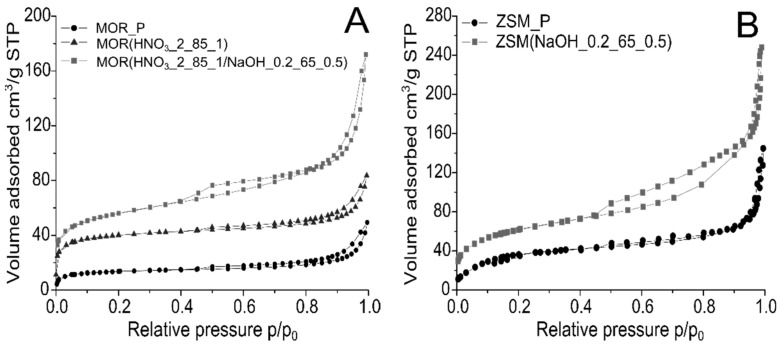
Comparison of N_2_ adsorption/desorption isotherms for parent and modified mordenite (**A**) and ZSM-5 (**B**).

The confirmation of the generation of the secondary pore system in zeolites marked as ZSM(NaOH_0.2_65_0.5) and MOR(HNO_3__2_85_1/NaOH_0.2_65_0.5) are N_2_ adsorption/desorption isotherms (Figure 2).

The microporous character of parent materials, besides the determined textural parameters (Table 3), are also confirmed also by the low temperature nitrogen adsorption isotherms. They assumed the type I shape, according to the IUPAC (International Union of Pure and Applied Chemistry) classification. The mesoporous character of MOR(HNO_3__2_85_1/NaOH_0.2_65_0.5) is confirmed by the shape of the nitrogen adsorption/desorption isotherm, which combines the type I and type IV (micro and mesoporous characters) (Figure 2A). This is in line with the textural analysis, where a significant increase in the specific surface area and mesopore volume was observed. The N_2_ adsorption/desorption isotherm shape of the ZSM-5 zeolite (Figure 2B) is a combination of type I and IV, which is characteristic for materials with a hierarchical system of pores, where both micro- and mesopores are present.

Summing up, the generation of an additional system of mesopores for mordenite and the ZSM-5 zeolite resulted in an increase in the adsorption capacity.

In summary, the proposed methods of modifying materials proved to be effective only in the case of materials treated with 2 M HNO_3_ and 0.2 M NaOH solutions. The best textural parameters were obtained for MOR(HNO_3__2_85_1/NaOH_0.2_65_0.5). Further sorption studies were therefore limited to materials referred to as MOR_P and MOR(HNO_3__2_85_1/NaOH_0.2_65_0.5), ZSM_P and ZSM(NaOH_0.2 M_65_0.5).

### 3.3. State of Silicon and Aluminum in and Modified Zeolite Materials

**Figure 3:**
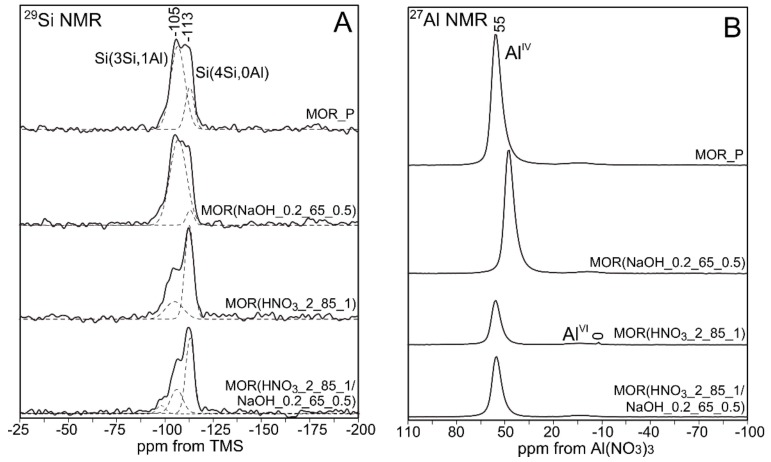
(**A**) ^29^Si MAS NMR and (**B**) ^27^Al MAS NMR spectra of MOR_P and its modified variants.

**Figure 4:**
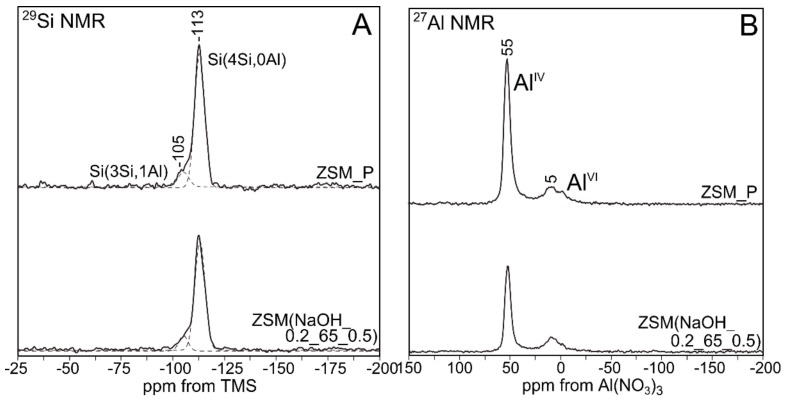
(**A**) ^29^Si MAS NMR and (**B**) ^27^Al MAS NMR spectra of ZSM_P and its modified variants.

The ^29^Si MAS NMR and ^27^Al MAS NMR spectra of parent and modified zeolites are presented in Figure 3 and Figure 4.

NMR studies make it possible to complete information on the environment and coordination of aluminum and silicon atoms in the zeolite crystal lattice.

In Figure 3A, which presents the ^29^Si MAS NMR spectra of parent MOR_P and desilicated MOR (NaOH_0.2_65_0.5) samples, two intensive signals are observed. The one at −105 ppm indicates the presence of silicon atoms in a tetrahedral coordination with oxygen ([SiO_4_] tetrahedron), with one alumina atom Si(1Al) in the vicinity. The second one, at −113 ppm, can be attributed to [SiO_4_] tetrahedra accompanied only by silicon atoms Si(0Al) [25]. Similar signals were recorded for materials designated as MOR(HNO_3__2_85_1) and MOR (HNO_3__2_85_1/NaOH_0.2_65_0.5). However, in the case of mordenite treated only with the HNO_3_ solution, the decrease in intensity of the Si(1Al) signal can be observed. This can be directly connected to the removal of alumina from the zeolite structure. When comparing MOR(HNO_3__2_85_1/NaOH_0.2_65_0.5) to MOR(HNO_3__2_85_1), the ratio between Si(1Al) and Si(0Al) signals is unchanged, which confirms the hypothesis concerning the lack of preference for silicon extraction. It can be presumed that the presence of [AlO_4_]^−^ stabilizes silicon atoms that are in the vicinity via the repulsion of OH^−^ ions [19].

The aim of ^27^Al MAS NMR studies (Figure 4B) was to evaluate the effect of demetalation process on the development and/or removal of extra-framework aluminum atoms that are typically associated with Lewis centers. The analysis of the obtained results showed that in all samples, aluminum assumed a tetrahedral coordination (Al^IV^) and was linked to Al(OSi)_4_, as evidenced by the presence of an intense signal at about 55 ppm [26,27]. This signal can also be attributed to the penta-coordinated aluminum atoms [28,29]. According to other sources, this signal may also be associated with disordered, tetrahedrally coordinated aluminum atoms in extra-framework positions (EFAl) [30]. Bokhoven, on the other hand, suggested that it can also be connected with the presence of distorted, tetrahedrally coordinated aluminum atoms in the structure [31]. In addition, the treatment of parent material with the acid solution caused the appearance of a signal at about 5 ppm. This signal was associated with octahedral aluminum (Al^VI^) [32,33]. The absence of octahedrally coordinated alumina in the case of MOR(NaOH_0.2_65_0.5) is observed. The results of textural studies for this material indicate the inefficiency of the desilication process in generating secondary mesoporosity (Table 3). The effect of desilication on MOR(HNO_3__2_85_1) was a significant increase in the signal originating from Al^IV^.

When analyzing the ^29^Si MAS NMR spectra recorded for ZSM_P and ZSM(NaOH_0.2_65_0.5) zeolites (Figure 4A), a signal at −113 ppm, corresponding to Si(4Si,0Al) after desilication, can clearly be observed. Moreover, the Si(3Si,1Al) signal (−105 ppm) after desilication is constant. This is due to the fact that [SiO_4_] tetrahedra, in the vicinity of which aluminum is located, exhibit higher stability during desilication. Thus, the Si–O–Al bond appears to be less susceptible to hydrolysis that which occurs in the presence of OH^−^ ions. The negative charge of [AlO_4_]^−^ tetrahedra stabilizes neighboring silicon atoms via the electrostatic repulsion of OH^−^ groups.

An analysis of the ^27^Al MAS NMR results shows that it is the parent ZSM-5 zeolite that contains aluminum, both Al(IV) in tetrahedral positions, as well as octahedral (in small quantities) Al(VI). The desilication process has contributed to the loss of intensity of the signal coming from the tetrahedral Al(IV). It has to be noticed that no correlation has been observed for the loss of intensity of the Al(IV) tetrahedral aluminum band and the increase of intensity of the band related to Al(VI) octahedral aluminum. This is consistent with literature [34] and may be a result of a strongly distorted coordination of aluminum. In addition, it is worth noting that the parent ZSM-5 zeolite does not show a great tendency to remove aluminum from lattice positions.

### 3.4. IR Spectra of Parent and Modified Zeolites

**Figure 5:**
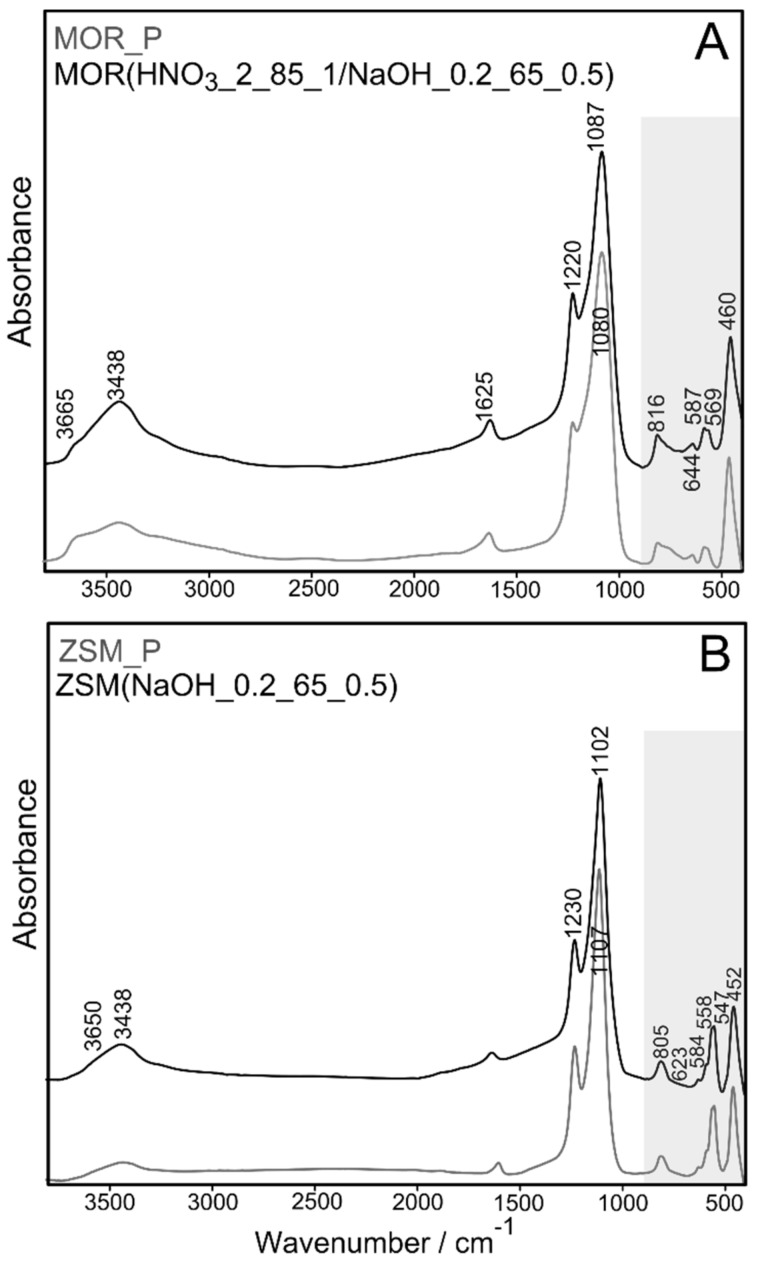
Mid-infrared (MIR) spectra of parent MOR_P and modified MOR(HNO_3__2_85_1/NaOH_0.2_65_0.5) (**A**) and ZSM_P and modified ZSM(NaOH_0.2_65_0.5) (**B**).

Figure 5 shows the FT-IR spectra of parent (ZSM_P and MOR_P) and their modified analogs (ZSM(NaOH_0.2_65_0.5) and MOR(HNO_3__2_85_1/NaOH_0.2_65_0.5)), registered in the full range of 4000–400 cm^−1^.

Due to the fact that the studied zeolites belong to the pentasil group, with a 5-1 secondary building unit, their structure consists of five-member rings, connected with an additional tetrahedron. The zeolite framework may be presented as a form of an overlapping system of tetrahedrons, interlinked by rings with varying numbers of members, including 10-, 12-, and 14-member rings [5]. An exemplary analysis of the OH group range (especially for the ZSM-5 zeolite) was discussed in the work [2]. In the range of 1450–400 cm^−1^ presented in Figure 5, bands associated with different types of vibrations in the zeolite structure can be distinguished. The entire range of 1200–450 cm^−1^ originates from oscillations of the Si–O–Si and Si–O–Al bonds occurring in [AlO_4_] and [SiO_4_] tetrahedra. Among them, the band located at 1100 cm^−1^ is attributed to asymmetrical vibrations of Si–O(Si,Al) groups. On the other hand, the band at about 450 cm^−1^ is associated with O–Si–O bending vibrations in tetrahedra. In the case of ZSM zeolite, desilication causes a shift of the aforementioned bands towards higher wavenumbers, which suggests decreasing silicon content in the materials. This is consistent with the conclusions drawn from the chemical composition studies of the investigated materials (Table 2). Moreover, the applied modification does not cause changes in the full width at half maximum (FWHM) of the discussed bands, which confirms that the samples preserve their crystallinity, as determined based on XRD analysis (Figure 1). In the case of MOR, inverse tendency was observed.

The range of 600–800 cm^−1^ indicates the presence of pseudo-lattice vibrations originating from over-tetrahedral structural units, mainly rings built of [SiO_4_] and [AlO_4_] tetrahedra (so-called ring vibrations) [35]. This range is particularly interesting because the zeolite structure is composed of rings with a different number of members. As mentioned above, the secondary building unit (SBU) of both analyzed materials is a 5-1 complex [34]. The main factors that affect the positions of these bands are the Si/Al ratio, number of ring members, as well as type of vibrations associated with the band and type of extra-framework cations [36]. The discussed materials belong to high- (ZSM-5) and medium-silica (MOR) zeolites. For higher Si/Al ratios, the bands-specific ring oscillations shift to higher frequencies. In the case of ring members, the inverse relationship occurs—for lower Si/Al ratios, the bands are located at lower wavenumber values. For all materials, bands that may be connected with five- and six-membered rings are observed in the range of 550–600 cm^−1^. The band connected with five-membered rings may be located in a complex envelope at 560 cm^−1^, but the intensity of these bands is low due to the unusual five-fold symmetry. On the other hand, the bands specific to the oscillation of six-membered rings are located at significantly higher wavenumbers, at about 600 cm^−1^, and can occur up to about 500 cm^−1^. The spectra in the analyzed wavenumber range have complex envelopes, which indicates that they are a superposition of several component bands [37,38]. Assuming that an increase in aluminum content results in the shift of ring-specific bands towards lower frequencies, it can be suggested that the band at about 750 cm^−1^ may be specific to four-membered rings (S4R). Furthermore, bands at 730 cm^−1^ are observed, which is most likely related to symmetrical vibrations of Si–O–Al.

### 3.5. Sorption of Heavy Metal Cations—Quantitative Studies

Since mordenite belongs to medium-silica zeolites, it had been thought that it exhibits a high ion-exchange capacity. This was confirmed by numerous studies devoted to this material [38]. For comparison, ZSM-5 zeolite, which is characterized by a high Si/Al ratio, has a poor ion-exchange ability and selectivity. In this respect, it is very different from medium- and low-silica zeolites [39,40].

**Figure 6:**
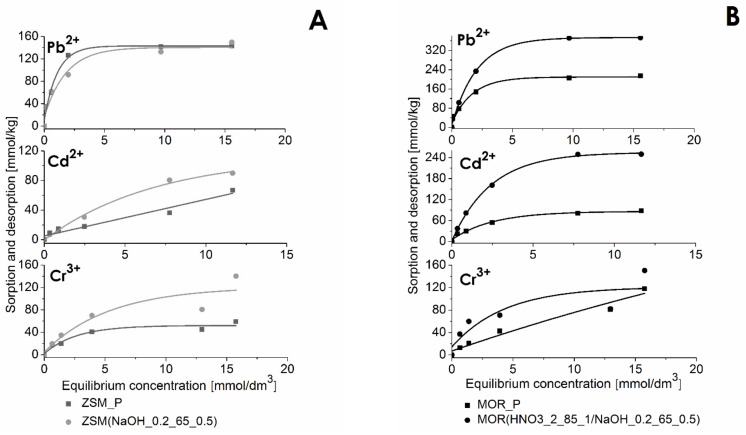
Results of sorption of heavy metal cations on ZSM_P and MOR_P and their modified analogs: (**A**) ZSM(NaOH_0.2_65_0.5) and (**B**) MOR(HNO_3__2_85_1/NaOH_0.2_65_0.5).

The AAS method was used to conduct the quantitative analysis of the sorption of heavy metal cations on the investigated materials (Table 4). This made it possible to determine the total concentration of ions immobilized in the zeolite structure via ion exchange and chemisorption. Parent MOR_P and ZSM_P materials as well as modified MOR(HNO_3__2_85_1/NaOH_0.2_65_0.5) and ZSM(NaOH_0.2_65_0.5) were selected for sorption studies. The amount of heavy metal cations sorbed on parent and modified materials as a function of the concentration of solutions is presented in Figure 6.

The obtained results show a significant increase in the amount of sorbed cations with an increase in solution concentration up to about 5 mmol/dm^3^. A further increase in solution concentration did not result in a noticeable increase in the effectiveness of sorption. As regards the analyzed cations, the maximum sorption capacities of ZSM_P and ZSM(NaOH_0.2_65_0.5) were Pb^2+^—143 and 149 mmol/kg, Cd^2+^—66 and 89 mmol/kg, Cr^3+^—59 and 140 mmol/kg, respectively; in the case of MOR_P and MOR(HNO_3__2_85_1/NaOH_0.2_65_0.5) the corresponding values were: Pb^2+^—214 and 373 mmol/kg, Cd^2+^—87 and 250 mmol/kg, Cr^3+^—118 and 150 mmol/kg. The investigated zeolites sorb different amounts of analyzed cations. The sorption capacity of ZSM-5, which is twice as low as that of mordenite, is directly related to its structure, which consists mainly of large numbers of silicon atoms (high Si/Al ratio) and, as a result, a small number of cations that can participate in ion exchange.

The expected consequence of demetalation is a change in the sorption capacity of zeolites. In the case of ZSM-5, desilication contributes to the reduction in the Si/Al ratio, increase in BET surface area, as well as to the formation of a mesopore system within the zeolite structure, which greatly facilitates the diffusion of ions into the intra-crystalline free space of zeolites. This may result in improved sorption properties of the modified materials. This is particularly evident after the sorption of Cd^2+^ and Cr^3+^ ions on the ZSM-5 zeolite and all heavy metal cations on mordenite. The increased concentration of adsorbed ions in the case of the modified materials may be related to the fact that these materials feature both micropores and mesopores in their structure. When assessing the sorption of divalent lead and cadmium cations, it can be seen that Pb^2+^ ions are sorbed in the largest amounts, both on ZSM-5 zeolite and mordenite. This may be related to the low hydration energy and relatively small ionic radii of these cations in aqueous solutions.

**Figure 7:**
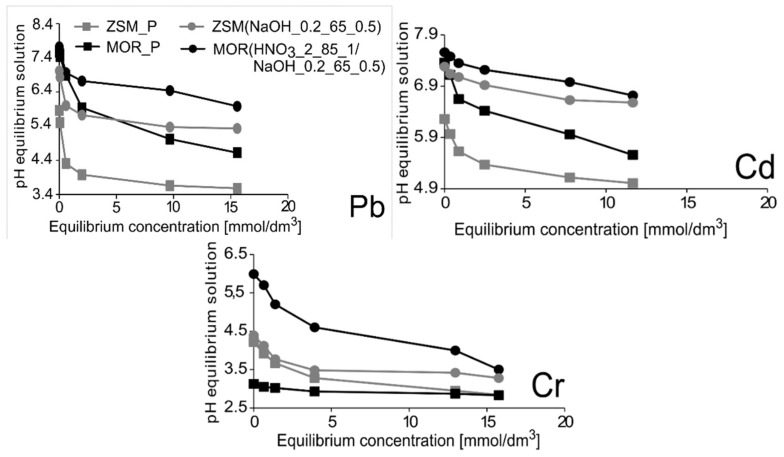
Results of pH values of equilibrium solutions with respect to equilibrium concentration of solutions containing heavy metal cations.

It is also worth mentioning that the sorption of individual cations depends on their chemical characteristics and on the pH of the equilibrium solutions. The pH parameter was measured for all parent solutions (from the concentration of 1 to 20 mmol/dm^3^) and for each solution left after the sorption process. The obtained results are presented in Figure 7. In the case of the Pb^2+^ ions, the pH of equilibrium solutions after sorption changed from 7.5 to 4.6. In such conditions the dominant ions were Pb(OH)^+^ cations [41]. Likewise, cadmium cations were present in the form of Cd(OH)^+^, dominating at higher pH values [42]. On the other hand, chromium ions tend to form water complexes, which limits the ability of this element to diffuse into the interior of micropores. The factor that can result in a relatively small amount of adsorbed chromium cations is the pH, which is maintained at 5.6–3.6. At this pH, Cr(OH)^2+^ ions are the dominant form in aqueous solution [43]. However, it should be noted that the amount of chromium sorbed on desilicated zeolites is three times as high, which may be due to the fact that chromium ions penetrate mesopores.

It is worth noting that all the materials were finally converted into the H^+^ form, which was used in sorption experiment. Experimental results clearly show that cation uptake considerably increases with increase in pH. This effect can be concluded in Figure 7. As mentioned above, both materials show large increase in the uptake, especially in case of MOR(HNO_3__2_85_1/NaOH_0.2_65_0.5). This level of increase in cation uptake could be explained by increase in solution pH alternatively to the chemical treatment, so in further studies cation uptake should be compared at a similar pH.

**Figure 8:**
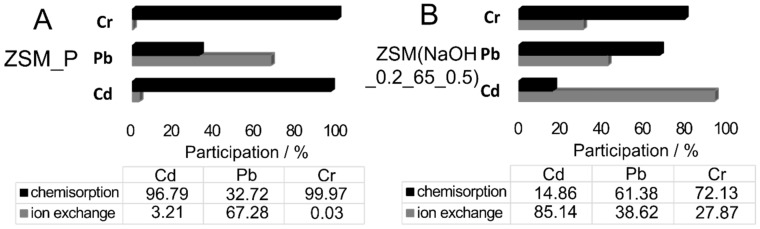

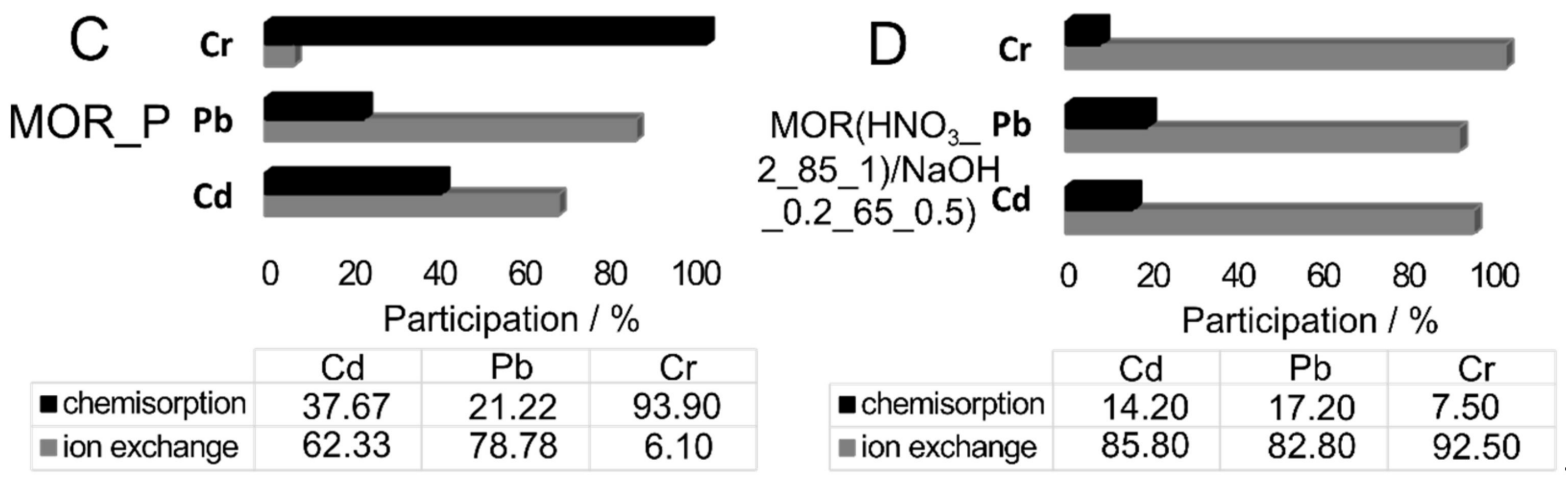
Comparison of chemisorption and ion-exchange processes involving the analyzed cations on studied materials ((**A**): ZSM_P; (**B**): ZSM(NaOH_0.2M65_0.5 M); (**C**): MOR_P and (**D**): MOR(HNO_3__2_85_1/NaOH_0.2 M_65_0.5) for the initial concentration of 20 mmol/dm^3^ of solution.

Based on the conducted sorption and desorption experiments, the proportions between the main mechanisms of immobilization were determined. The results are collected in Figure 8.

Comparable concentrations of cadmium ions were sorbed via ion exchange and chemisorption (only in the case of the ZSM-5 zeolite). On the other hand, in the case of lead ions, ion exchange was predominant (in the case of both zeolite types). The main mechanism of the immobilization of trivalent chromium ions was chemisorption, which involves the persistent binding of the sorbed cation on the outer surface of the zeolite. However, it is possible that some of these ions were not introduced into the aluminosilicate structure, and instead precipitated in the form of Cr(OH)_3_ [44]. The small contribution of ion exchange is confirmed by the fact that, for trivalent cations, ion exchange is generally poor [45]. It should nevertheless be noted that, contrary to what might be expected based on literature data, the amount of immobilized Cr^3+^ cations for all samples was clearly higher than that of divalent cations. The converse was true only in the case of modified mordenite. For the MOR_P sample, the dominant mechanism was still chemisorption, but the proposed modification caused a visible increase in the contribution of ion exchange, which leads to the conclusion that it has a positive effect on sorption processes.

### 3.6. Sorption of Heavy Metal Cations—Qualitative Research

#### Infrared Spectroscopy

**Figure 9:**
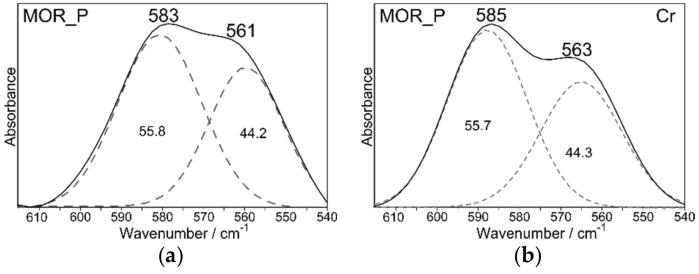

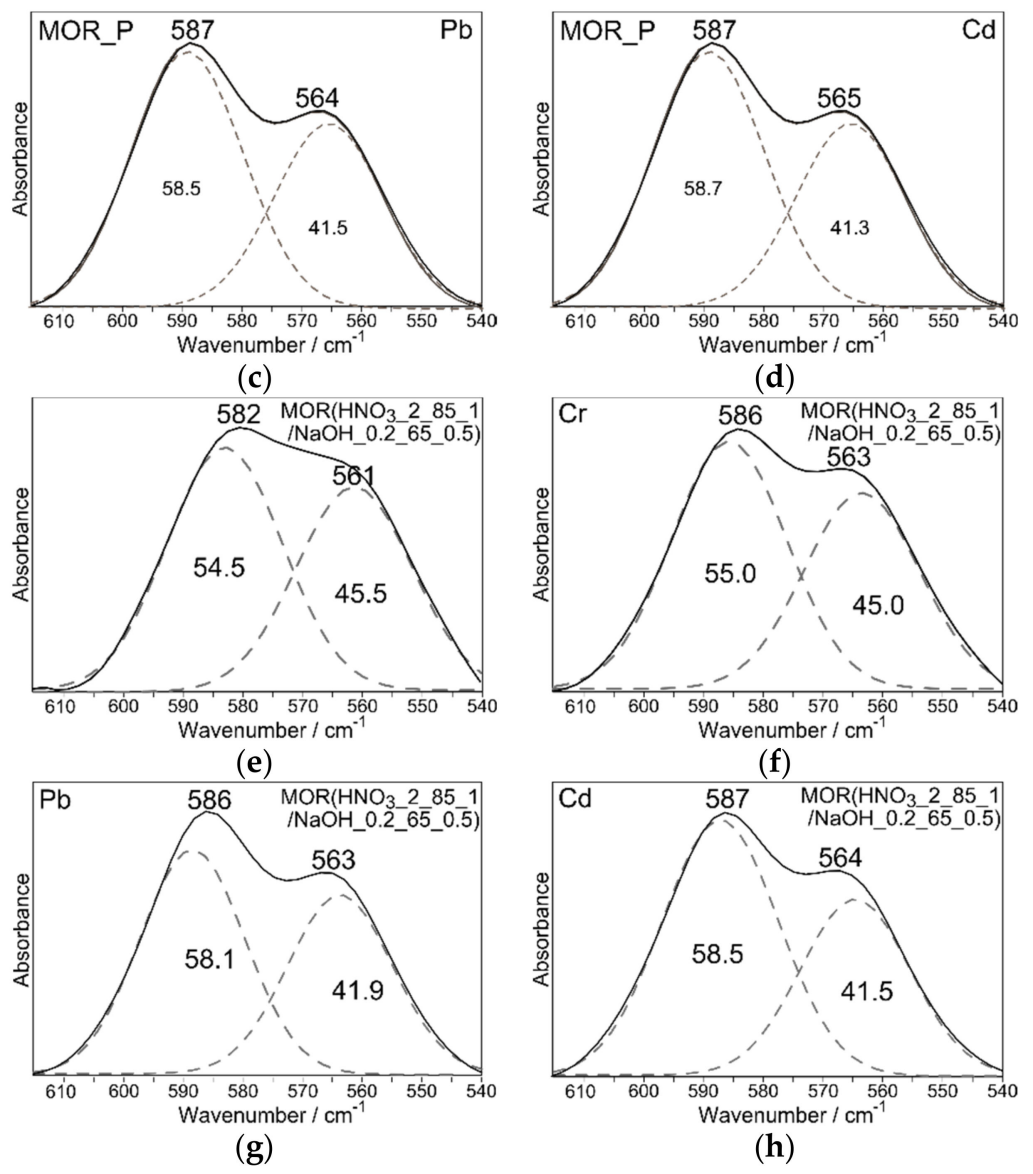
Decomposition of MIR spectra of MOR_P and MOR(HNO_3__2_85_1/NaOH_0.2_65_0.5) before (**a**,**e**) and after sorption of Pb^2+^ (**c**,**g**), Cd^2+^ (**d**,**h**) and Cr^3+^ (**b**,**f**) ions from an aqueous solution (C_0_ = 20 mmol/dm^3^).

**Figure 10:**
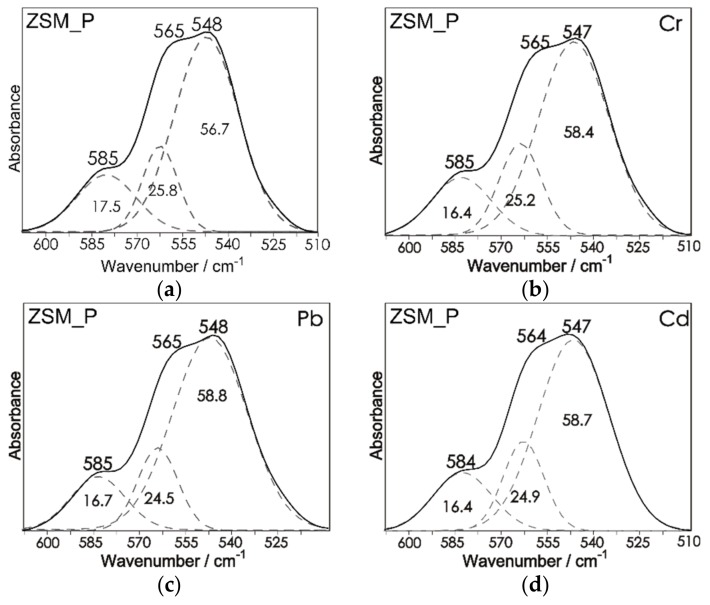

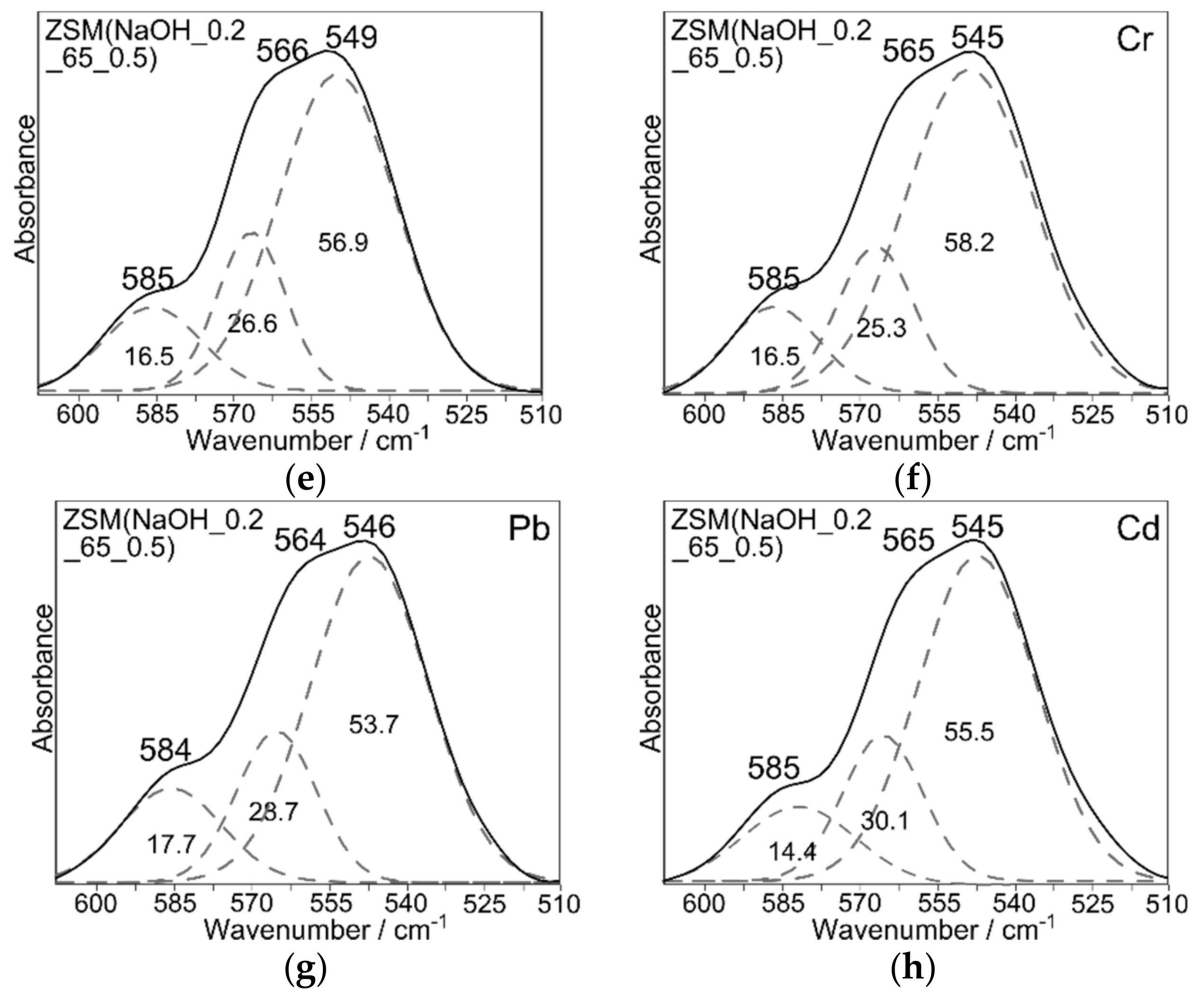
Decomposition of MIR spectra of ZSM_P and ZSM(NaOH_0.2_65_0.5) before (**a**,**e**) and after sorption of Pb^2+^ (**c**,**g**), Cd^2+^ (**d**,**h**) and Cr^3+^ (**b**,**f**) ions from an aqueous solution (C_0_ = 20 mmol/dm^3^).

The sorption of heavy metal cations on the studied zeolites should be accompanied by changes in IR spectra. The introduction of cations differing in atomic mass, charge, and ionic radius into the structure leads to a change in ring surroundings, affecting its symmetry and causing deformation, which results in a shift in the range of ring vibrations. Chemisorption process results in the formation of stable inner-sphere complexes. This is due to the fact that functional groups of aluminosilicate networks (mainly OH) form strong chemical bonds with the ion without hydration layer [46]. In zeolites, the ion-exchange process usually dominates over chemisorption, but not in all cases. Figure 9 and Figure 10 show the spectra recorded in the mid-infrared region after the sorption of Pb^2+^, Cd^2+^, and Cr^3+^ cations (C_0_ = 20 mmol/dm^3^) on the ZSM_P and MOR_P zeolites.

More detailed assessment of intensity changes is possible after decomposing the spectra into component bands. The intensity of the bands is expressed on a percentage scale, i.e., as the integral intensity of the band at 564 cm^−1^ (for mordenite) and 585 cm^−1^ (for ZSM-5) to the sum of band intensities.

Introduction of foreign cations into the structure results in a slight increase in the intensity of bands in the range of 585–550 cm^−1^. Bands that occur in that frequency range are typical of five-membered rings. However, the most noticeable changes are observed both for parent and modified mordenite, at about 564 cm^−1^ in regard to [38]. Changes in that range may result from the varied nature of cations that coordinate hydroxyl groups, i.e., their behavior in an aqueous environment. The changes after sorption of Pb^2+^ and Cd^2+^ cations are very similar. The exception is for Cr^3+^ cations, which have a strong tendency to undergo hydrolysis and to form various types of aqueous complexes in a solution.

Interpretation of the results has been presented only for mordenite and may be transferred to the ZSM-5 zeolite. In the case of ZSM-5 zeolite (both for parent and modified samples), changes in the range of 510–615 cm^−1^ are not so visible. This is related to the fact that the ZSM-5 zeolite is characterized by a lower Si/Al ratio.

## 4. Conclusions

The obtained results show that the sequential treatment of zeolites with alkaline and acid solutions is a very effective method of generating a secondary mesopore system. The modifications yielded materials with a large surface area (S_BET_), a well-developed surface of mesopores, and preserved microporosity. The modified materials proved to be better sorbents of heavy metal cations than their precursors, as confirmed by AAS studies. The NMR and IR spectra revealed changes in zeolite structures induced by both demetalation processes and the sorption of heavy metal cations. The performed modification of zeolite materials contributed to the improved effectiveness of the sorption of heavy metal cations. The applied modification increased both the porosity and sorption capacity, which results in the increase of the ion exchange rate of both zeolites, especially in the case of chromium ions sorption.

## Figures and Tables

**Table 1:** Detailed procedures and conditions of zeolite modifications with sample designations.

Type of Modification	Conditions	Notation
-	-	ZSM_P
three-fold ion-exchange into NH_4_^+^	0.5 M NH_4_NO_3_, T = 60 °C, t = 1 h	MOR_P
desilication/ion-exchange into NH_4_^+^	0.2 M NaOH, T = 65 °C, t = 0.5 h	ZSM(NaOH_0.2_65_0.5)
		MOR(NaOH_0.2_65_0.5)
dealumination/ion-exchange into NH_4_^+^	2 M HNO_3_, T = 85 °C, t = 1 h	MOR(HNO_3__2_85_1)
	4 M HNO_3_, T = 85 °C, t = 1 h	MOR(HNO_3__4_85_1)
dealumination/desilication/ion-exchange into NH_4_^+^	0.2 M NaOH, T = 65 °C, t = 0.5 h	MOR(HNO_3__2_85_1/NaOH_0.2_65_0.5)
		MOR(HNO_3__4_85_1/NaOH_0.2_65_0.5)

**Table 2:** Chemical composition of the studied materials.

Sample	SiO_2_	Al_2_O_3_	Na_2_O	K_2_O	CaO	TiO_2_	Fe_2_O_3_	Others	Si/Al
ZSM_P	97.011	2.760	0.025	0.032	0.012	0.113	0.036	0.036	29.020
MOR_P	90.542	8.504	0.099	0.053	0.070	0.544	0.073	0.115	9.033
ZSM(NaOH_0.2_65_0.5)	96.150	3.330	0.024	0.015	0.020	0.350	0.049	0.062	24.496
MOR(NaOH_0.2_65_0.5)	88.956	9.884	0.090	0.050	0.053	0.441	0.136	0.252	7.736
MOR(HNO_3__2_85_1)	95.445	3.892	0.032	0.022	0.019	0.452	0.021	0.157	20.805
MOR(HNO_3__4_85_1)	95.705	3.260	0.045	0.046	0.065	0.504	0.063	0.312	24.901
MOR(HNO_3__2_85_1/NaOH_0.2_65_0.5)	94.342	5.090	0.021	0.022	0.019	0.452	0.021	0.033	16.020
MOR(HNO_3__4_85_1/NaOH_0.2_65_0.5)	93.505	5.586	0.045	0.036	0.043	0.423	0.056	0.306	14.201

**Table 3:** Chemical and textural parameters of the studied materials.

Sample	Si/Al_XRF_	Si/Al_NMR_	Crystallinity (%)	S_BET_ (m^2^/g)	S_EXT_ (m^2^/g)	V_micro_ (cm^3^/g)	V_meso_ (cm^3^/g)
ZSM_P	29.020	33.089	100	433	39.5	0.180	0.166
MOR_P	9.033	7.902	100	374	12.1	0.170	0.164
ZSM(NaOH_0.2_65_0.5)	24.496	26.563	94	475	108.1	0.170	0.342
MOR(NaOH_0.2_65_0.5)	7.736	5.839	90	376	10.7	0.170	0.166
MOR(HNO_3__2_85_1)	20.805	17.985	95	392	19.0	0.170	0.175
MOR(HNO_3__4_85_1)	24.901	23.524	92	377	18.6	0.160	0.166
MOR(HNO_3__2_85_1/NaOH_0.2_65_0.5)	16.020	14.025	87	421	92.2	0.150	0.292
MOR(HNO_3__4_85_1/NaOH_0.2_65_0.5)	14.201	13.325	82	335	46.2	0.140	0.223

**Table 4:** Sorption results of heavy metal cations on mordenite and ZSM-5 zeolite as a function of concentration.

Zeolite	C_0_	Pb^2+^			Cd^2+^			Cr^3+^		
		S	IE	Ch	S	IE	Ch	S	IE	Ch
**MOR_P**	1	45.31	36.53	8.78	21.74	18.08	3.66	12.96	5.75	7.21
	2	77.26	71.03	6.23	39.70	22.55	17.15	21.05	7.15	13.90
	5	147.35	115.81	31.54	54.06	34.82	19.24	42.54	7.92	34.62
	15	205.49	156.82	48.67	80.74	50.21	30.53	81.22	7.71	73.51
	20	213.88	168.49	45.39	87.46	54.51	32.95	117.99	7.20	110.79
**MOR(HNO_3__2_85_1/NaOH_0.2_65_0.5)**	1	34.85	30.15	4.70	36.97	30.32	6.65	37.18	31.99	5.19
	2	102.61	88.04	14.57	81.43	77.71	3.72	59.80	44.67	15.13
	5	233.59	188.48	45.11	161.15	145.77	15.38	70.77	70.16	0.61
	15	371.28	274.40	96.88	249.35	205.01	44.34	82.25	71.83	10.42
	20	372.95	308.89	64.06	249.90	214.23	35.67	150.35	139.01	11.34
**ZSM_P**	1	34.75	23.87	10.88	8.95	1.88	7.07	17.63	0.03	17.60
	2	60.18	54.63	5.55	14.31	2.66	11.65	19.78	0.02	19.76
	5	126.47	91.51	34.96	17.83	2.24	15.59	41.08	0.01	41.07
	15	141.62	94.84	46.78	36.20	2.44	33.76	44.95	0.02	44.93
	20	143.30	96.41	46.89	66.73	2.14	64.59	59.00	0.02	58.98
**ZSM(NaOH_0.2_65_0.5)**	1	33.16	8.91	24.25	6.77	4.58	2.19	19.50	17.49	2.01
	2	62.02	31.82	30.20	11.73	8.47	3.26	34.72	19.11	15.61
	5	91.63	33.68	57.95	30.51	20.90	9.61	70.09	21.01	49.08
	15	132.47	55.71	76.76	80.40	69.01	11.39	80.80	25.53	55.27
	20	149.73	57.82	91.91	89.86	76.51	13.35	140.31	39.10	101.21

Symbols: C_0_—initial concentration of the solution (mmol/dm^3^); S—total sorption (mmol/kg); IE—ion exchange (mmol/kg); Ch—chemisorption (mmol/kg).
